# Leaf Monoterpene Emission Limits Photosynthetic Downregulation under Heat Stress in Field-Grown Grapevine

**DOI:** 10.3390/plants10010181

**Published:** 2021-01-19

**Authors:** Massimo Bertamini, Michele Faralli, Claudio Varotto, Maria Stella Grando, Luca Cappellin

**Affiliations:** 1 Center Agriculture Food Environment (C3A), University of Trento, Via. E. Mach 1, 38010 San Michele all’Adige, Italy; stella.grando@unitn.it; 2 Research and Innovation Centre, Fondazione Edmund Mach, Via E. Mach 1, 38010 San Michele all’Adige, Italy; claudio.varotto@fmach.it (C.V.); luca.cappellin@unipd.it (L.C.); 3 Department of Chemical Sciences, University of Padua, Via Marzolo 1, 35131 Padova, Italy

**Keywords:** leaf monoterpene emission, heat stress tolerance, chlorophyll fluorescence, photosynthesis, grapevine

## Abstract

Rising temperature is among the most remarkably stressful phenomena induced by global climate changes with negative impacts on crop productivity and quality. It has been previously shown that volatiles belonging to the isoprenoid family can confer protection against abiotic stresses. In this work, two *Vitis vinifera* cv. ‘Chardonnay’ clones (SMA130 and INRA809) differing due to a mutation (S272P) of the DXS gene encoding for 1-deoxy-D-xylulose-5-phosphate (the first dedicated enzyme of the 2C-methyl-D-erythritol-4-phosphate (MEP) pathway) and involved in the regulation of isoprenoids biosynthesis were investigated in field trials and laboratory experiments. Leaf monoterpene emission, chlorophyll fluorescence and gas-exchange measurements were assessed over three seasons at different phenological stages and either carried out in in vivo or controlled conditions under contrasting temperatures. A significant (*p* < 0.001) increase in leaf monoterpene emission was observed in INRA809 when plants were experiencing high temperatures and over two experiments, while no differences were recorded for SMA130. Significant variation was observed for the rate of leaf CO_2_ assimilation under heat stress, with INRA809 maintaining higher photosynthetic rates and stomatal conductance values than SMA130 (*p* = 0.003) when leaf temperature increased above 30 °C. At the same time, the maximum photochemical quantum yield of PSII (F_v_/F_m_) was affected by heat stress in the non-emitting clone (SMA130), while the INRA809 showed a significant resilience of PSII under elevated temperature conditions. Consistent data were recorded between field seasons and temperature treatments in controlled environment conditions, suggesting a strong influence of monoterpene emission on heat tolerance under high temperatures. This work provides further insights on the photoprotective role of isoprenoids in heat-stressed *Vitis vinifera*, and additional studies should focus on unraveling the mechanisms underlying heat tolerance on the monoterpene-emitter grapevine clone.

## 1. Introduction

High-temperature stress (heat stress, HS) is one of the main environmental conditions affecting crop physiology and productivity [1,2]. According to the fifth assessment report of the Intergovernmental Panel on Climate Change, further increases in annual temperatures are expected in the next future [3]. In the Mediterranean viticulture, temperature above optimum has been linked to yield reduction and to a decrease in grape and wine quality [4], owing to impaired physiological performances and phenological hastening [5]. It is generally accepted that HS in grapevine occurs when leaf heat dissipation capacity is smaller than the total absorbed solar energy and, under limited water availability, following reduced leaf transpiration and accumulation of latent heat [6]. In addition, the degree of heat stress damage in grapevine depends on both maximum diurnal temperature and the duration of daily HS, with significant physiological downregulation in days with prolonged HS hence bringing further complexity in the assessment of the genotypic heat tolerance [4].

High temperatures can have direct harmful effects associated with physical damage to tissues or indirect effects linked with changes in plant metabolism [1,2]. One of the main consequences of HS often coupled with the presence of intense solar radiation is the excessive production of reactive oxygen species (ROS), which leads to oxidative stress [7]. ROS accumulation significantly reduces photosynthetic capacity in grapevine with carbon metabolism of the stroma and photochemical reactions in thylakoid membranes considered as the primary sites of injury at high temperatures [8]. Early work suggested that HS reduces or even interrupts the activity of photosystem II (PSII), leading to significant reduction in CO_2_ assimilation and leaf source capacity [9,10].

Many traits have been associated with increased HS tolerance in grapevine such as elevated transpiration rate per unit of leaf area, cellular turgidity maintenance through osmotic adjustment and modification in the antioxidant system to restore the cellular redox balance and homeostasis [1,11]. Additionally, the synthesis of volatile organic compounds (VOCs) has often been related with increased HS tolerance in several species [12]. In particular, terpenes are the main VOCs produced by plants under HS, and high temperature has been shown to be the major driver of terpene biosynthesis [13,14]. The photoprotection role of terpenes has been extensively reported, and a tight link between HS tolerance and terpene emission has been often observed [15,16]. However, most available literature focuses on the role of isoprene, i.e., the most emitted plant hydrocarbon into the atmosphere [17]. Grapevine is not an isoprene-emitting species, although some varieties are known to produce high levels of volatile monoterpenes [18]. While numerous studies have indicated that monoterpenes may play an important regulatory role under plant stress [13,14,15,16], their biological functions in grapevine are still poorly understood [19]. Indeed, the emission capacity of monoterpenes from vine leaves in aromatic varieties and their potential role on heat stress tolerance has never been extensively explored. Therefore, in this work, two field-grown Chardonnay clones with contrasting monoterpene emission rates were monitored over three consecutive years. This study aims: (i) to evaluate the effects of HS on key grapevine leaf physiological traits such as chlorophyll fluorescence and gas-exchange; (ii) to assess the variation in emission of volatile monoterpenes from leaves of two cv. Chardonnay clones (SMA130 and INRA809) differing for a mutation of the DXS gene involved in the regulation of isoprenoids biosynthesis and under different degrees of HS; (iii) to determine a potential relationship between tolerance to HS and monoterpene emission.

## 2. Results

### 2.1. Experiments 2017

**Figure 1:**
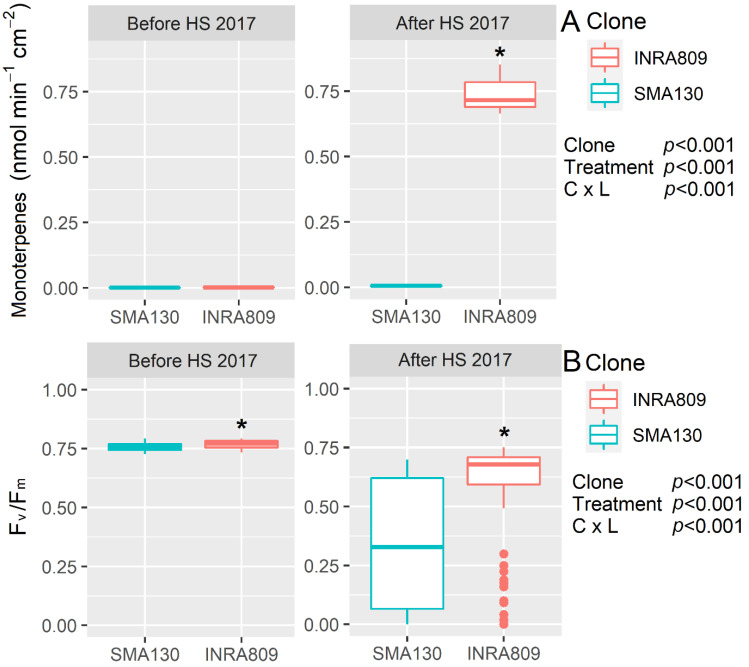
Monoterpenes emission (**A**) and maximum quantum yield of photosystem II in dark-adapted samples (F_v_/F_m_) (**B**) analyzed in INRA809 and SMA130 before and after heat stress (HS) (*n* = 20–30). Data were collected in 2017 and subjected to a two-way ANOVA. ANOVA output is shown in the figure and for each trait. Asterisks show significant differences between clones according to *t*-test.

Monoterpene emission was not detectable before HS for both the SMA130 and INRA809 clones, while a significant increase in the emission (0.75 nmol min^−1^ cm^−2^, *p* < 0.001) was observed after HS for INRA809 (Figure 1). Similarly, SMA130 had lower F_v_/F_m_ values before and after HS when compared to INRA809 (*p* < 0.001), with the latter showing sustained F_v_/F_m_ after heat stress compared to the relative control before the HS treatment (*p* < 0.001).

### 2.2. Experiments 2018

**Figure 2:**
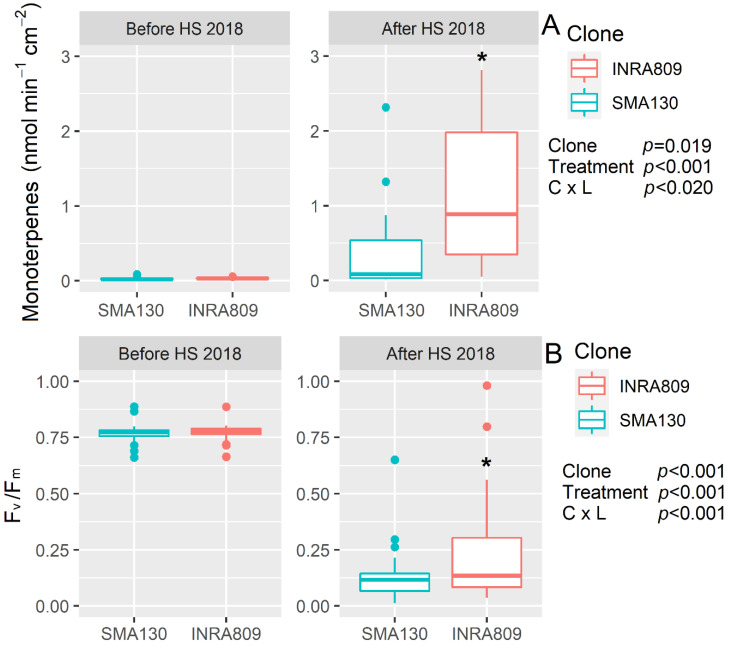
Monoterpenes emission (**A**) and maximum quantum yield of photosystem II in dark-adapted samples (F_v_/F_m_) (**B**) analyzed in INRA809 and SMA130 before and after HS (*n* = 20–30). Data were collected in 2018 and subjected to a two-way ANOVA. ANOVA output is shown in the figure and for each trait. Asterisks show significant differences between clones according to *t*-test.

In 2018 and before HS, no significant monoterpene emission was observed in either clone. After HS (*p* < 0.001), INRA80 showed a higher monoterpene emission (*p* = 0.019) than SMA130 (Figure 2). HS significantly reduced F_v_/F_m_ (*p* < 0.001), while no differences were recorded before HS and between SMA130 and INRA809 for F_v_/F_m_. However, lower F_v_/F_m_ was observed in SMA130 under HS when compared to INRA809 (*p* < 0.001).

### 2.3. Experiments 2019

**Figure 3:**
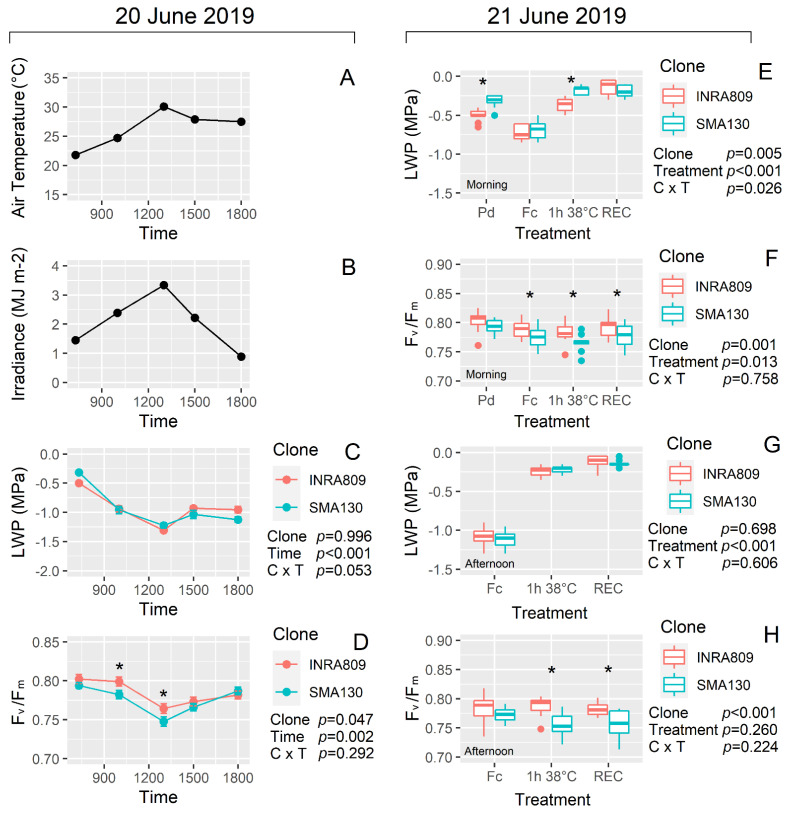
Diurnal changes in air temperature, irradiance, leaf water potential (LWP) and maximum quantum yield of photosystem II in dark-adapted samples (F_v_/F_m_) collected on 20 June 2019 (**A**–**D**) and for INRA809 and SMA130. (**E**,**F**) represent the LWP and F_v_/F_m_ response at different temperature conditions imposed in controlled conditions and in leaves collected in the morning of 21 June 2019 (Pd, pre-dawn; Fc, field condition; 1 h 38 °C, conditions imposed in the growth cabinet; REC, recovery in the growth cabinet at 24 °C). (**G**,**H**) represent the LWP and F_v_/F_m_ response at different temperature conditions imposed in controlled conditions and in leaves collected in the afternoon of 21 June 2019 (Pd, pre-dawn; Fc, field condition; 1 h 38 °C, conditions imposed in the growth cabinet; REC, recovery in the growth cabinet at 24 °C). Data are means (*n* = 9–10) ± standard error of the means (SEM). Data were collected in 2019 and subjected to a two-way ANOVA. ANOVA output is shown in the figure and for each trait. Asterisks show significant differences between clones according to *t*-test.

On 20 June 2019, the average temperature during LWP and fluorescence measurements increased from 23 °C in the early morning to up to 30 °C at 13:00. Similar trends were observed for solar radiation (Figure 3A,B). *In vivo* measurements showed a significant drop in LWP during the hottest portion of the day (*p* < 0.001), while no significant differences for LWP between clones (*p* = 0.996) were observed (Figure 3C). Similar trends were recorded for dynamic daily F_v_/F_m_, with significant drops during the warmest hours (*p* = 0.002). On the contrary, differences were recorded for F_v_/F_m_ between clones at 10:00 and 13:00 with INRA809 exhibiting lower photoinhibition levels than SMA130 (Figure 3D).

Analysis carried out on the 21 June under controlled conditions revealed similar trends in F_v_/F_m_ between cultivars with INRA809 showing higher values than SMA130 under HS and recovery conditions both in the morning and the afternoon (*p* < 0.001 for both) (Figure 3F and H). In the morning, significant variation was observed for LWP between the two cultivars at Pd and 1 h 38 °C (*p* = 0.005) with SMA130 having less negative LWP than INRA809 (Figure 3E and G).

**Figure 4:**
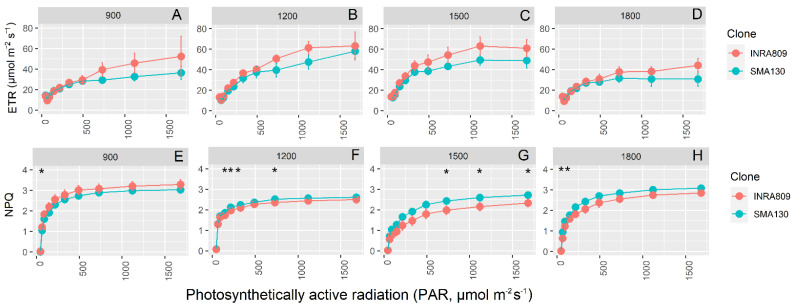
Rapid light curves for INRA809 and SMA130. Electron transport rate (ETR, **A**–**D**) and non-photochemical quenching (NPQ, **E**–**H**) collected in four periods (900, 1200, 1500 and 1800) over the day. For environmental data, see Figure 3A,B. Data are means (*n* = 4–6) ± standard error of the means (SEM). Asterisks (*) show significant differences between clones according to *t*-test for each light level and between clones.

RFLC data highlights significant diurnal variation for ETR and NPQ, with higher ETR values at 12:00 and 15:00 when compared to 9:00 and 18:00 (Figure 4). INRA809 did not yield significantly higher ETR than SMA130 at any of the light conditions applied, although some trends (*p* < 0.1) are present for data collected at 15:00. In contrast, SMA130 had steadily higher NPQ values than INRA809, in particular at 12:00 and 15:00 (*p* < 0.001). Consistent variation between the clones was observed at 15:00, with SMA130 having higher NPQ at the highest light intensity and compared to INRA809.

**Figure 5:**
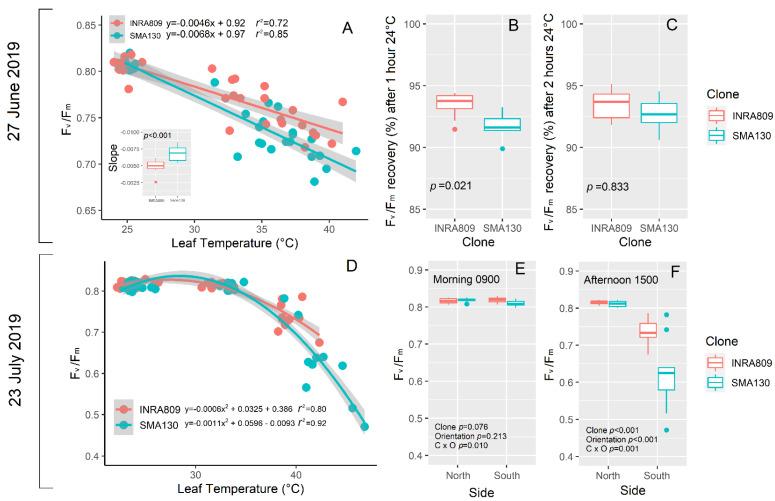
Relationship between leaf temperature (°C) and F_v_/F_m_ in INRA809 and SMA130 (**A**). A linear regression was fit for each individual, and the slope was subjected to one-way ANOVA (**A**). In B and C, the % of F_v_/F_m_ recovery compared to 25 °C leaf temperature condition is shown after 1 h (**B**) and 2 h (**C**). Data were collected on 27 June 2019 (n = 5–10) and subjected to one-way ANOVA. In (**D**), the relationship between leaf temperature (°C) and F_v_/F_m_ in INRA809 and SMA130 is shown. A second-order polynomial curve was fit to the data. In (**E**,**F**), the F_v_/F_m_ collected either in the morning (**E**) and in the afternoon (**F**) in INRA809 and SMA130 is shown. Analysis was carried out on both the side of the raw (*n* = 10), and data were subjected to two-way ANOVA (output in the graph).

Under in vivo HS conditions (27 June), a linear regression explained the negative relationship between F_v_/F_m_ and leaf temperature for both the clones (Figure 5A). However, the slope of the regression (*n* = 5) was significantly more negative for SMA130 than INRA809. INRA809 displayed a better recovery after 1 h at 24 °C (*p* = 0.021) when compared to SMA130, while no differences were observed after 2 h (Figure 5B,C).

On 23 July, a non-linear polynomial fit explained the relationship between F_v_/F_m_ and leaf temperature (Figure 5D). Increasing leaf temperature led to lower F_v_/F_m_ values in SMA130 than INRA809, although the increase in leaf temperature was more severe in SMA130 than INRA809. Analysis carried out on differently exposed sides of the raw revealed no significant differences between clones and the raw side in the morning (Figure 5E). On the contrary, a significant reduction in PSII activity was observed in the afternoon (*p* < 0.001) and on the south side only, with lower F_v_/F_m_ in SMA130 than INRA809 (*p* < 0.001) (Figure 5F).

**Figure 6:**
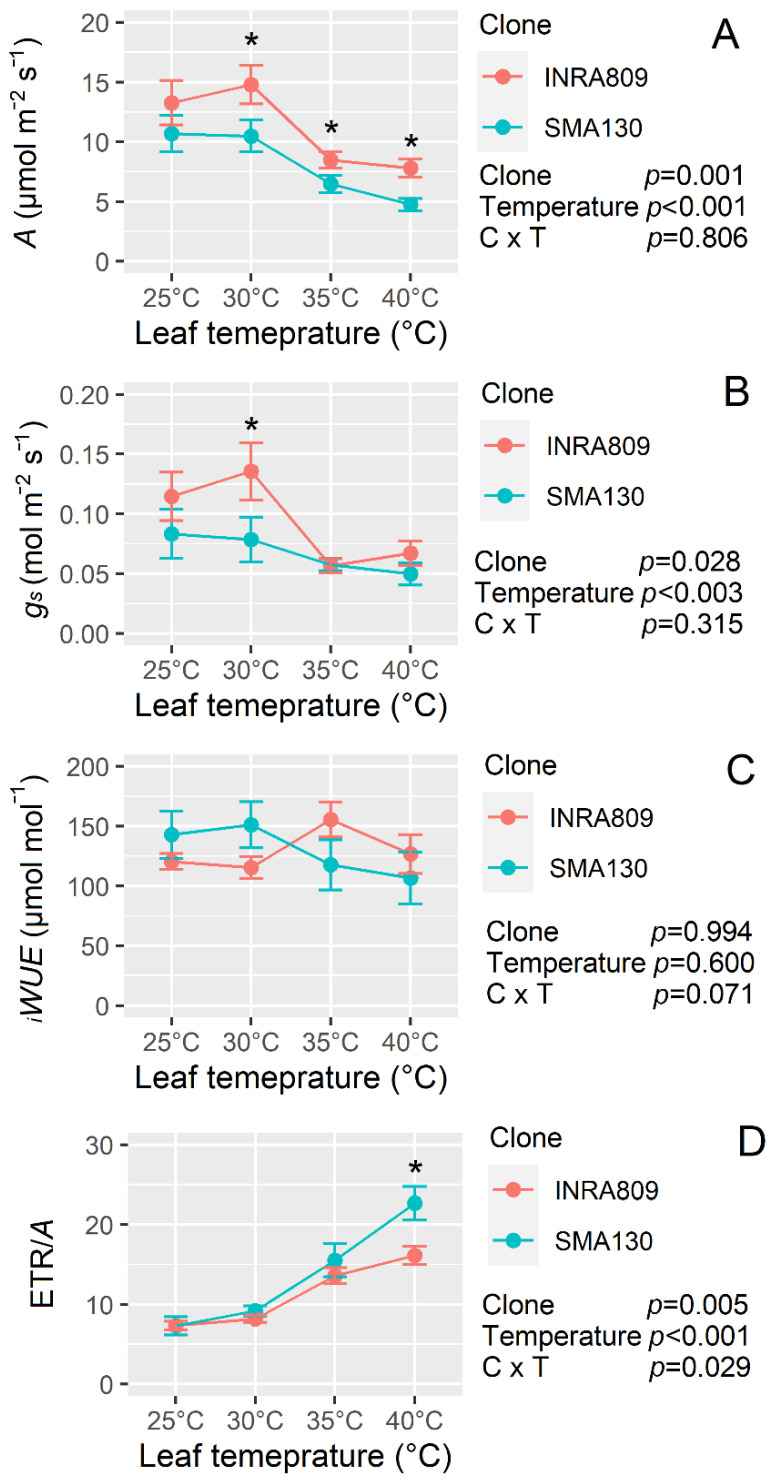
Temperature response of two grapevine clones (INRA809 and SMA130) for CO_2_ assimilation (*A*, **A**), stomatal conductance (*g_s_*, **B**), intrinsic water-use efficiency (*_i_WUE*, **C**) and the ratio between ETR and *A* (ETR/*A*, **D**). Data were collected with a Licor6400 and at increasing leaf temperatures (25, 30, 35, 40 °C). Data are means (*n* = 5) ± standard error of the means (SEM). Data were collected on 6 August 2019 and subjected to two-way ANOVA (output in the graph). Asterisks show significant differences between clones according to *t*-test.

Temperature response curves revealed a significant negative effect of increasing temperature on all gas-exchange traits (*p* < 0.001) apart from *_i_WUE* (*p* = 0.600) (Figure 6). Indeed, increasing temperature significantly reduced *A* and *g_s_* (Figure 6A and B)*,* while a greater ETR/*A* ratio was observed (Figure 6D). INRA809 had higher *A* when compared to SMA130, and this was significant at 30 °C, 35 °C and 40 °C (*p* < 0.001). Higher *g_s_* values were recorded for INRA809 and compared to SMA130 (*p* = 0.028) but at 30 °C only. The ETR/*A* was higher in SMA130 than INRA809 at 40 °C (*p* = 0.005).

## 3. Discussion

### 3.1. Heat Stress Negatively Influences Grapevine Physiology

Grapevine is generally considered well adapted to Mediterranean environmental conditions characterized by restricted water availability and high temperatures, especially during summer [20]. The results of our work, however, suggest a generalized and significant negative effect of HS on physiological performances of grapevine.

Although with different degrees between the pair of Chardonnay clones, HS-induced monoterpenes emission in both 2017 and 2018. The temperature dependence of monoterpene emission in plants has been previously shown [12,21], and several enzymes appear to be involved in monoterpene biosynthesis under stress [22]. Monoterpene emission has been often linked to thermotolerance in several species [12,23], either by improving membrane heat resistance [23] or indirectly by acting as antioxidants and quenching free-radicals [24]. Our work suggests that the HS experienced at the time of analyses triggered defensive mechanisms likely due to a marked oxidative stress.

Previous work reported F_v_/F_m_ as an excellent indicator of HS tolerance in grapevine [9], thus, validating the approach used in our study. Higher susceptibility of *Vitis vinifera* varieties to HS was previously observed when compared to wild grape and hybrids [9], and the acceptor side of the PSII was less damaged by heat than the donor side or the reaction center in grape leaves. Indeed, over three independent seasons characterized by different degrees of HS and growing conditions (pot and field), a significant reduction in the maximum quantum yield of PSII was evident for both the clones tested accompanied by detrimental effects on water status (diurnal reduction in LWP during the hottest part of the day) and slow recovery. The reduction in F_v_/F_m_ under heat stress may be associated with several processes such as (1) modification in the PSII super-complex hampering energy transfer from antenna complexes to the PSII reaction center [25]; (2) alteration in the oxygen-evolving complex activity [26]; (3) increased photo-protective regulatory mechanisms [27]; (4) inhibition of the photosynthetic electron transport chain [28]. These mechanisms may act jointly and can be affected by the intensity and length of HS: further work is, therefore, required to dissect the mechanisms underlying F_v_/F_m_ reduction in grapevine under heat stress.

Similarly, rapid increase in leaf temperature led to a reduction in *A* that was significant at 35 °C and 40 °C. In other species acclimated to Mediterranean environments, similar thermal sensitivity thresholds of *A* were found [29], although genotypic variation was previously recorded [30]. Our data suggest that either a reduction in photochemistry (F_v_/F_m_) or an increase in photorespiration (increase in ETR/*A* ratio) are induced under HS in grapevine, although impairment in Rubisco activity and mesophyll diffusion cannot be excluded [31].

Under high temperatures, an increase in *g_s_* and transpiration has been often observed for several species [32]. Indeed, as temperatures rise, the increase in evaporative cooling associated with higher *g_s_* can maintain leaf temperature to optimal levels for photosynthesis [33,34] or even prevent leaf temperatures from reaching a harmful threshold for leaf survival. In our work, *g_s_* was reduced under high leaf temperatures, potentially following an ABA-induced stomatal closure owing to an increase in leaf-to-air vapor pressure deficit [35]. Nevertheless, a reduction in *g_s_* was already observed in some fruit crops subjected to HS and was previously associated with an adaptation to minimize the risk of xylem embolism [29]. We speculate that long-term adaptation to concomitant low water availability and heat stress might prioritize reductions in *g_s_* to avoid cavitation in grapevine, at least at the environmental conditions applied in this work.

### 3.2. Monoterpene Emission Induces Photoprotective Mechanisms in Grapevine under Heat Stress

Monoterpene emission has been previously shown to induce heat stress tolerance in several species [16,23,36]. For instance, *Quercus ilex* leaves fumigated with monoterpenes had a lower decline in photosynthesis compared to non-fumigated leaves when exposed to high temperatures [23]. Similarly, transgenic *Arabidopsis* mutants constitutively emitting monoterpene (ocimene) had higher *A* and projected leaf area when grown under HS conditions and compared to Col-0 [16]. In our work and consistent with previous findings in other species, the monoterpene emitter INRA809 steadily maintained high F_v_/F_m_ after HS in several experiments followed by fast post-stress recovery. In addition, non-photochemical energy dissipation was often higher in SMA130 than INRA809, consistent with other reports where isoprenoid-emitting ecotypes were compared [37]. Differences in NPQ have been often related to changes in carotenoids with a severe increase in antheraxanthin and zeaxanthin and a consequently higher oxidation of xanthophylls under stress conditions [37]. Similarly, native non-isoprenoid-emitting species are also characterized by higher levels of zeaxanthin and NPQ [38], suggesting that isoprenoid emission (in this case monoterpene) can protect photosynthesis at least under the stress conditions applied in this work. Similar results were reported by [15], comparing a wild-type and isoprene-emitting tobacco mutants, showing that isoprene emission maintains PSII stability at high temperatures by providing a homogeneous distribution of the light-absorbing centers and a stable thylakoid membrane stiffness. This is in line with the higher *A* found in INRA809 at higher leaf temperature and compared to SMA130 accompanied by a lower ETR/*A* ratio at 40 °C. However, ETR/*A* ratio increased in both clones under HS suggesting that the rate of reducing power in excess of that used for photosynthesis was partitioned to other sinks, with INRA809 likely to partition excessive electrons to the monoterpenes biosynthetic pathway. In addition, a higher ETR/*A* ratio for SMA130 at 40 °C under stress suggests an increase in distribution of electrons to alternative sinks at high temperatures that may indicate greater photorespiratory losses [39].

A more negative slope in the regression of leaf temperature to F_v_/F_m_ was observed for SMA130 compared to INRA809, suggesting that increasing leaf temperature led to a more rapid reduction in photochemical efficiency. The fact that the monoterpene emitter INRA809 maintained higher F_v_/F_m_ for a similar leaf temperature to SMA130 may further confirm an induced thermotolerance of the emitter clone. Consistent with this work, F_v_/F_m_ declined much less in isoprene-emitting tobacco plants subjected to environmental stress [15,40]. However, data collected on the 23 July 2019 highlighted a much higher increase in leaf temperature for identical environmental conditions in SMA130 than INRA809. This increase in leaf temperature can be mainly attributed to a reduced leaf evaporative cooling in SMA130 following restricted transpiration rates at high temperature and hence VPD levels. Gas-exchange data confirm that *g_s_* was higher in INRA809 than SMA130, but only at a leaf temperature of around 30 °C, thus indicating a higher stomatal sensitivity of SMA130 when leaf temperature reached above 40 °C. Developing water stress cannot be the cause of the differences in transpiration rates as TDR analysis carried out in 2019 showed a field volumetric water content between 40 and 45% at 30cm soil depth (data not shown). VOC emissions have been previously shown to be partially involved in *g_s_* responses to environmental stimuli [16]. For instance, isoprene-emitting capacity can induce fast stomatal closure and elevated stomatal sensitivity to soil water stress, mainly to avoid tissue dehydration [41]. The conservative behavior of isoprene-emitter *Arabidopsis* in [16] was associated with a strategy to increase the internal isoprene concentration (due to its high volatility) under stress conditions and to enhance its potential beneficial effect, which is minimized under low concentrations [42]. Conversely, monoterpene-emitting species are more common in xeric habitats than isoprene-emitter species, and a non-conservative strategy is advantageous for maximizing nutrient capture and for successful colonization of habitats with significant fluctuations in resource availability [43]. Therefore, it is not surprising that indications of a lower stomatal sensitivity to HS (and high VPD) are present in the monoterpene emitter INRA809 compared to SMA130, as already shown in *Arabidopsis* [16]. The generally significant HS tolerance coupled with a higher transpiration rate under severe HS conditions and high VPD in INRA809 might be considered a combination of preferable traits to overcome severe heat waves, at least in situations where irrigation is available. Further work is needed, however, to (1) test our hypothesis on water and heat stress interaction tolerance for INRA809 and to (2) dissect the molecular mechanisms involved in the higher HS tolerance displayed by the monoterpene emitter clone.

## 4. Materials and Methods

### 4.1. Plant Material and Growing Conditions

The experiments were carried out over three consecutive years in 2017, 2018 and 2019. In 2017 and 2019, data were collected from field-grown material, while in 2018, cuttings from the field vines were used for a pot experiment. Two clones of Chardonnay (SMA130 and INRA809) differing in their aromatic pattern were compared [44]. The ENTAV-INRA^®^ 809 clone (INRA809) was selected in 1995 in Saône-et-Loire (Bourgogne, France) and is an aromatic musquè clone, characterized by a medium productive level and high sugar and acidity level, and is associated with fine and balanced wines. The SMA^®^ 130 clone (SMA130) was selected in 1978 at the Agricultural Experimental Station of San Michele all’Adige (Trento, Italy). It is characterized by a good level of productivity, a good level of organic acids and sugars, and the absence of terpenic compounds in the bunch and the must. Compared to other clones of Chardonnay, the Muscat character of INRA 809 has been attributed to a mutation (S272P) in 1-deoxy-D-xylulose 5-phosphate synthase 1 (VvDXS1), a key enzyme of the methylerythritol phosphate pathway for isoprenoid precursor biosynthesis in grapevine [45,46,47].

For the field experiments (2017 and 2019), the two clones were grown in the vineyard located at the agricultural site of the Fondazione Edmund Mach at San Michele all’Adige (46°11′28′′N, 11°8′11′′E, 232 m of altitude). The vineyard was planted in 2004 in a field with a 15 to 20% slope. The vineyard has west exposure with a calcareous skeletal soil, a loam-limestone texture, 15% clay, low organic substance and a balanced content of nutritive elements. Density of planting was 5600 plants ha^−1^ and vines were pruned to a guyot system. Temperature and irradiance were monitored with a weather station 50 m away from the field site.

For the pot experiment carried out in 2018, cuttings (*n* = 10) were taken from the experimental vineyard at bud dormancy on 8 January 2018. The material was then moved for 2 weeks in an environmentally controlled growth chamber at 4 °C to avoid inhibition of the budburst. Subsequently, cuttings were standardized at two buds, the basal bud was removed and was immersed in a rooting hormone solution for 30 days in a solution of 2 g of indole-3-butyric acid (IBA), 1 g of Potassium Hydroxide, hydrogen chloride (HCL) 37% and Sodium hydroxide (NaOH) to reach a pH 7 in 1 L of water. Cuttings were then planted in 1.7 L pot containing a mix of soil:sand:peat:vermiculite (3:1:3:3) *v/v* and placed in a greenhouse at 25/20 °C day/night temperature, with a 16 h photoperiod, relative humidity (RH) of 70 ± 10% and natural light conditions in a fully randomized design. On 4 April, the plants were pruned at the sixth leaf to stimulate the growth of the secondary shoots.

### 4.2. Environmental Conditions and Stress Application

#### 4.2.1. Field Experiments 2017

For each clone and before harvest (11 September 2017), sun-exposed leaves (*n* = 20) on the 12th node of the shoot facing the south side of the canopy were collected pre-dawn along with the petiole. Collected leaves were placed in deionized water and immediately moved to the laboratory where 1.5 cm at the base of the petiole was re-cut under water to avoid cavitation (this procedure was followed for petiole re-cutting in all the following experiments). Samples were placed in test tubes containing fresh de-ionized water and moved to a growth chamber (Climacell 707, BMT Medical Technology s.r.o, Brno, Czech Republic). The HS treatment was applied the same day in the same growth chamber and consisted in the following temperature steps: an acclimation temperature of 25 °C for 1.30 h, followed by a temperature increase to 52 °C for 2 h. RH and photosynthetic active radiation (PAR) were kept constant at 70% and 270 µmol m^−2^ s^−1^, respectively. Total monoterpene emission and leaf chlorophyll fluorescence were measured before and immediately after the HS treatment.

#### 4.2.2. Pot Experiments 2018

Plants in the greenhouse were moved under natural conditions (June 2018) for one week. Each experimental day, only plants belonging to the same clone (*n* = 5 per day per clone) were tested in order to avoid plant–plant communication during the experiment. Temperature, RH, PAR and HS protocol were kept as in 2017. Monoterpene emission and leaf chlorophyll fluorescence were measured before and immediately after the HS treatment.

#### 4.2.3. Field Experiments 2019

The experiments were carried out on four different days: 20, 21 and 27 June and 23 July. For all experiments, sun-exposed leaves (*n* = 10) for each clone placed on the central node of the shoot (8–10° node) were selected.

On 20 June, the analysis was carried out at four different times: 7:30, 10:00, 13:00, 15:00 and 18:00. Measurements of leaf water potential and chlorophyll fluorescence were taken in vivo. The same day, rapid light curves were collected at 9:00, 12:00, 15:00, 18:00.

On 21 June, the samples were taken at 07:30 (Pd), 09:30, 10:00, 11:30 for the morning analysis and at 14:00, 15:00 and 16:00 for the afternoon analysis. Leaves were collected as detailed above and immediately moved to the laboratory where the control chlorophyll fluorescence and leaf water potential were recorded. After that, the petiole was re-cut, samples were placed in test tubes containing fresh de-ionized water and moved to a growth chamber (Climacell 707, BMT Medical Technology s.r.o, Brno, Czech Republic). The HS treatment was applied the same day in the growth chamber and consisted of the following temperature steps: an acclimation temperature of 25 °C for 1 h (Fc), followed by a temperature increase to 38 °C for 1 h (1 h 38 °C) and recovery period (24 °C) for 1 h (REC). RH and PAR were kept constant at 70% and 270 µmol m^−2^ s^−1^, respectively.

On 27 June, analyses were carried out at 08:30, 11:30 and 14:00 in vivo. Before each sampling and at each time of the day, the area used for chlorophyll fluorescence was tagged on each leaf, and leaf temperature was measured with an infrared thermometer (Handheld Infrared Laser Thermometer, 62 MAX+, FULKE Corporation, Everett, Washington USA). Subsequently, the leaves used for analysis were sampled after 15:00 and placed in tubes containing deionized water and immediately moved to the laboratory. After that, the petiole was re-cut and leaves were placed in a growth chamber for a recovery period at 24 °C. Measurements of chlorophyll fluorescence were taken after 1 and 2 h from the start of the recovery period.

On 23 July, leaves were tagged at 09:00 and 15:00 on either the sun-exposed side of the canopy (south side) and the shaded ones (north side). Before each sampling and at each time of the day, the area used for chlorophyll fluorescence was tagged on each leaf, and leaf temperature was measured with an infrared thermometer (Handheld Infrared Laser Thermometer, 62 MAX+, FULKE Corporation, Everett, Washington, USA)

### 4.3. Physiological Assessments

#### 4.3.1. Monoterpene Emission Analysis

Total monoterpene emission assessments were carried out in 2017 and 2018. In 2017, the experiments were carried out on leaves (*n* = 20) collected from the field for each clone and on the same day as the chlorophyll fluorescence measurement. The leaves were immediately placed in deionized water and inside a growth chamber (Climacell 707, BMT Medical Technology s.r.o, Brno, Czech Republic)

In 2018, plants were placed within a growth chamber (as above) over two consecutive days and enclosed using a Teflon PFA bag. In each experimental day of 2018, only samples belonging to the same clone were measured in order to avoid plant–plant communication during the experiments.

For both measurements (2017 and 2018), a commercial gas calibration unit instrument (Ionicon Analytik GmbH, Innsbruck, Austria) was employed to generate zero air, which was delivered to each bag at a constant flow of about 350 sccm. A PEEK capillary delivered the enclosure air to a commercial PTR-ToF-MS 8000 instrument (Ionicon Analytik GmbH, Innsbruck, Austria). An overflow allowed excess air to exit the enclosure. The temperature treatments described in the above sections were performed after two hours, during which monoterpene emission stabilized at the constitutive level. During the measurements, an automated inlet switching system allowed the PTR-ToF-MS to cycle between each enclosure every 12 min. The instrumental conditions were 550 V drift voltage, 2.25 mbar drift pressure and 110 °C drift tube temperature, resulting in an E*/N* ratio of about 140 Townsend (Td; 1 Td = 10^−17^ V cm^2^). *E* corresponds to the electric field strength, and *N* corresponds to the gas number density. The ToF sampling conditions were as follows: 0.1 ns sampling time per channel of ToF acquisition, amounting to 350,000 channels for a mass spectrum ranging up to *m/z* = 400. Every ToF spectrum is the sum of about 28,600 acquisitions lasting 35 μs each, thus resulting in a time resolution of 1 s. In order to avoid any memory effects, the spectra belonging to the first 60 s after enclosure switching were discarded and the remaining spectra were considered for further analysis. Count losses due to the ion detector dead time were accounted for using a method based on Poisson statistics [48]. Mass calibration, noise reduction, baseline removal, and peak intensity extraction were carried out according to [49]. Absolute monoterpene headspace concentrations expressed in parts per billion by volume (ppbv) were estimated from C10H17+ (parent protonated peak) and C6H9+ (main fragment) peak intensities as explained in [50], using a rate coefficient of 2 × 10^−9^ cm^3^/s. Leaf emissions expressed in nmol·min^−1^·cm^−2^ were estimated from ppbv concentrations considering the measured incoming air flux into the encloser and the enclosed leaf area.

#### 4.3.2. Chlorophyll Fluorescence

For all experiments, chlorophyll fluorescence was measured on the adaxial leaf surface using a PAM 2000 fluorimeter (Walz, Effeltrich, DE). The Handy PEA fluorimeter (Hansatech Instrument Ltd., Norfolk, UK) was used for the measurements carried out on 23 July 2019. All leaves were dark-adapted before any measurement for 30 min. The maximum quantum yield of PSII efficiency in dark-adapted samples (F_v_/F_m_) was calculated as F_v_/F_m_ = (F_m_ − F_o_)/F_m_, where F_o_ and F_m_ represent the minimum and maximum (after a saturation pulse of 6000 µmol m^−2^ s^−1^ PAR) fluorescence, respectively [27]. On 20 June, rapid chlorophyll fluorescence light-response curves (RFLC) were carried out with the PAM 2000 and using a pre-installed software protocol. For each RFLC, the actinic light was increased in a stepwise manner (20 s for each step) after an initial dark-adapted measurement. Light levels were 46, 66, 91, 148, 216, 330, 491, 730, 1119 and 1681 µmol m^−2^ s^−1^ PAR. For each PAR level, the light-adapted quantum yield of PSII (F_q_’/F_m_’) was derived from the maximum light-adapted florescence (F_m_’) and the minimum fluorescence (F_s_) as F_q_’/F_m_’ = (F_m_’ − F_s_)/F_m_’. The electron transport rate (ETR) was then calculated as ETR = PAR × F_q_’/F_m_’ × 0.5 × 0.84, where 0.5 and 0.84 are electron partitioning between PSII and PSI and leaf absorbance, respectively. Non-photochemical quenching (NPQ) was estimated as NPQ = (F_m_ − F_m_’)/F_m_’ [51,52].

#### 4.3.3. Leaf Water Potential

Leaf water status was monitored through analysis of leaf water potential (LWP) on the 20 and 21 June 2019. Briefly, sampled leaves were placed into a plastic bag and quickly positioned inside a Scholander pressure chamber (Model 3000 Scholander Plant Water Status Console, ICT International, Armidale, Australia). Readings of LWP were taken and expressed as MPa.

#### 4.3.4. Gas-Exchange Analysis

To assess the impact of rapid increases in leaf temperature on leaf gas exchange traits, a LiCor Li6400XT fitted with a 6400-40 2 cm^2^ leaf cuvette (Li-Cor Inc., Lincoln, NE, USA) was used. Data were collected in vivo in the field on 6 August 2019 between 8:00 and 15:00. Leaves (*n* = 5) were subjected to four leaf temperatures of 25, 30, 35 and 40 °C. In the Li-Cor cuvette, all parameters (leaf CO_2_ assimilation at saturating light—*A* and stomatal conductance—*g_s_*) were collected at an ambient [CO_2_] of 400 μmol mol^−1^. PAR was maintained at a saturating level of 1800 μmol m^−2^ s^−1^ with a 10:90 blue:red light and a flow rate of 500 mol s^−1^. Intrinsic water-use efficiency was calculated as *_i_WUE* = *A*/*g_s_*. All data were collected after the sample achieved steady state, and F_s_ and F_m_’ were additionally recorded by ensuring a light-saturating pulse of 8000 μmol m^−2^ s^−1^. Electron transport rate was calculated as above, and the ratio between ETR and CO_2_ assimilation at saturating light was calculated as ETR/*A*.

#### 4.3.5. Statistical Analysis

Statistical analyses were carried out with Rstudio (R Core Team 2018, PBC, Boston,USA, http://www.rstudio.com/). All data were checked for normality and homoscedasticity through visual assessment of distribution and residuals versus fitted values. When skew distribution was present, data were log-transformed. Relationships between variables were assessed following linear regression or 2^nd^ order polynomial. Data were then subjected to two-way ANOVA or one-way ANOVA depending on factor number. Means separation was carried out via Student’s *t*-test.

## 5. Conclusions

The results of this work provide evidence of a tight relationship between monoterpene emitting capacity and physiological responses to HS in Chardonnay clones. Although reduction in PSII activity was evident for both the clones under HS, INRA80 maintained higher F_v_/F_m_ throughout a range of environmental conditions and faster recovery after stress. The trends in response to HS were similar for both clones but higher *A*, *g_s_* and lower leaf temperature were observed in INRA809 compared to SMA130. While monoterpene emission can be considered a preferable trait under HS, additional desirable traits were detected in INRA809 (e.g., higher evaporative cooling under developing HS). This work sheds new light on the relationship between VOC emission and heat tolerance in an economically important crop such as grapevine, and further work will help to understand the molecular and physiological bases of monoterpene-induced HS tolerance.

## Data Availability

The data presented in this study are available on request from the corresponding author.
